# Dietary Phytochemicals in Cardiovascular Disease Prevention and Management: A Comprehensive Review

**DOI:** 10.1002/fsn3.70872

**Published:** 2025-09-03

**Authors:** Muhammad Tayyab Arshad, M. K. M. Ali, Sammra Maqsood, Ali Ikram, Md. Sakhawot Hossain, A. I. Aljameel, Ammar AL‐Farga, Kodjo Théodore Gnedeka

**Affiliations:** ^1^ Functional Food and Nutrition Program, Faculty of Agro‐Industry Prince of Songkla University Songkhla Thailand; ^2^ Department of Physics, College of Sciences Imam Mohammad Ibn Saud Islamic University (IMSIU) Riyadh Saudi Arabia; ^3^ National Institute of Food Science and Technology University of Agriculture Faisalabad Faisalabad Pakistan; ^4^ University Institute of Food Science and Technology The University of Lahore Lahore Pakistan; ^5^ Department of Nutrition and Food Technology Jashore University of Science and Technology Jashore Bangladesh; ^6^ Department of Biochemistry, Faculty of Science University of Jeddah Jeddah Saudi Arabia; ^7^ Togo Laboratory: Applied Agricultural Economics Research Team (ERE2A) University of Lomé Lome Togo

**Keywords:** antioxidant, carotenoids, phytochemicals, polyphenols

## Abstract

Cardiovascular disease (CVD) is the leading cause of death worldwide and a major public health issue. According to recent World Health Organization (WHO) estimates, CVD causes approximately 17.9 million deaths annually, accounting for 32% of global mortality. Prevention strategies that are dependent on dietary and lifestyle changes are becoming increasingly popular despite advances in pharmaceutical treatments. There has been a growing interest in phytochemicals and bioactive compounds in plants, as well as their potential to aid cardiovascular health and reduce the risk of cardiovascular disease. However, there is no comprehensive synthesis of their mechanisms, clinical relevance, and bioavailability. This review attempted to fill this gap by evaluating the therapeutic value of phytochemicals for the prevention and management of CVD. Dietary sources and classifications of cardioprotective phytochemicals, including phytosterols, carotenoids, alkaloids, and polyphenols, are extensively discussed in this review. The article goes in‐depth on how they act through many different functions, such as antioxidant, anti‐inflammatory, lipid‐modulating, and endothelial‐protective activities. Beyond describing the relationship between phytochemicals and broader dietary trends and lifestyle patterns, this article addresses their influences on bioavailability, including food matrix effects and gut microbiota. This review highlights the therapeutic significance of phytochemicals and their possible incorporation into standard treatments based on epidemiological, clinical, and mechanistic studies. Finally, this highlighted the need for future studies to fully exploit phytochemicals in the prevention and treatment of CVD, as well as the current issues in this regard.

## Graphical abstract

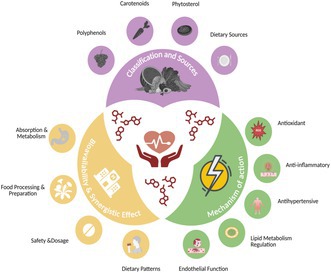

## 1 Introduction

Over a third of every annual death, approximating 17.9 million individuals, results from cardiovascular disease (CVD) (Chong et al. 2024; Goh et al. 2024). Owing to subpar healthcare centers and poor accessibility of preventive care measures, the situation is particularly precarious in low‐ and middle‐income countries (Einarson et al. 2018; Hinton et al. 2018). The most frequent forms of cardiovascular disease (CVD), including atherosclerosis, ischemic heart disease (IHD), hypertension, and stroke, have multifactorial etiologies involving the environment, heredity, and lifestyle options (Balakumar et al. 2016; Faizal et al. 2021). Although diagnostic equipment and drug interventions have progressed significantly, there remain some limitations to the current treatments. While efficacious, statins, antihypertensives, and antiplatelet drugs have costs, a history of side effects, and reduced compliance (Mohamed et al. 2022; Alradwan et al. 2024).

Appel (2017) and Martínez‐González et al. (2019) stated that the treatment‐based model failed to meet with success in reducing the rising prevalence of CVD; thus, there has been a global shift towards prevention‐based strategies, particularly those related to lifestyle modifications and food. Many studies have demonstrated the cardioprotective benefits of the Mediterranean diet, which consists of a high intake of plant foods, nuts, and whole grains (Guasch‐Ferré et al. 2018; Albert et al. 2002). Critical functional composites with the ability to prevent and manage CVD have emerged from phytochemicals, a diverse group of plant‐based biologically active composites (Dillard and German 2000; Liu 2013). Leitzmann (2016), Cicero and Colletti (2016), and Islam et al. (2021) demonstrated that various phytochemicals, including phytoestrogens, phytosterols, carotenoids, flavonoids, and polyphenols, exert antioxidant, anti‐inflammatory, lipid‐lowering, and vasodilatory activities. Later studies also demonstrated the additional antioxidant and cytotoxic potential of phytochemical‐rich plant extracts in their anti‐cancer activity against cancer cell lines. Roy, Prasad, and Ghosh (2023) demonstrated that the methanolic extract of Tupistra nutans exhibited broad antioxidant activity and selective cytotoxicity towards liver cancer cells HepG2, wherein it was ascribed to its high content of unsaturated fatty acids, diosgenin, and vitamin E. In addition, Roy, Prasad, Priya, et al. (2023) reported that the methanolic extract of *Rauvolfia serpentina* leaves inhibited the proliferation and migration of HepG2 and HeLa cancer cells while also inducing reactive oxygen species (ROS) generation, mitochondrial injury, and DNA injury. These mechanistic results indicate the broader therapeutic potential of food phytochemicals, in addition to cardiovascular protection. ROS are recognized as the cause of endothelial dysfunction and atherogenesis, and anthocyanins and flavonoids neutralize ROS (Betteridge 2000; Adegbola et al. 2017; Darawsha et al. 2021).

Phytochemical‐rich foods have been demonstrated to reduce cardiovascular risk in several trials. Regular intake of nuts has been reported by Altamimi et al. (2020) and Ellsworth et al. (2001) to reduce LDL (low‐density lipoprotein) cholesterol and enhance endothelial function. Grape juice, which is high in polyphenols, exerts anti‐inflammatory effects in patients with coronary artery disease (Albers et al. 2004). Conversely, cocoa flavonoids increase the levels of high‐density lipoprotein (HDL) and decrease oxidized LDL (Khan et al. 2012). These findings support the belief that phytochemicals added as part of a balanced diet might have an effect on maintaining heart wellness (Liu 2004; Hasler 2002). Additionally, emerging evidence suggests that phytochemicals counteract injury by modulating multiple molecular pathways, including the control of NO production, suppression of NF‐κB activation, and inhibition of lipid peroxidation (Islam et al. 2021; Haines et al. 2024). Soybean isoflavones and other phytoestrogens possess estrogenic activity and can assist postmenopausal women with healthy lipid profiles and vessel compliance (Azadbakht 2007; Mangano et al. 2013).

Additionally, evidence exists that plant‐based diets with high bioactive content can reduce systemic inflammatory markers such as C‐reactive protein and interleukin‐6. These markers have been linked to the progression of cardiovascular diseases (Biswal et al. 2013; Campbell et al. 2006). Clinical guidelines and public health policies might benefit from the addition of phytochemicals as pharmaceutical alternatives (Pagliaro et al. 2015; Nahar et al. 2020). Dwyer et al. (2018) and Liu (2013) reported that standardized dosing, bioavailability, and long‐term safety remain as challenges to be addressed. Despite these qualifications, adding phytochemicals to preventative strategies offers a compelling and cost‐effective means of reducing the global burden of CVD, which in turn enhances cardiovascular health and overall well‐being (Maqsood et al. 2025). However, there is growing evidence for the cardioprotective effects of phytochemicals, and no comprehensive synthesis that incorporates their complex molecular mechanisms, clinical efficacy, bioavailability considerations, and potential interactions with conventional therapies exists. Furthermore, variations in study design and the lack of standardization of dosing hinder the extrapolation of current data to clinical practice and public health policy. This review aims to fill these gaps by critically evaluating recent advances and presenting an integrated summary of the role of dietary phytochemicals in cardiovascular disease prevention and management. Based on epidemiological evidence, clinical trials, and mechanistic studies, the present review aims to critically analyze the existing data regarding the role of phytochemicals in cardiovascular health. In this article, we underscore the significance of specific phytochemicals and foods in which they are present as a measure for preventing cardiovascular disease via dietary means.

The main purpose of this research is to gather the latest scientific evidence on the role of phytochemicals in food, as far as their prevention and management of cardiovascular diseases is concerned. Existing clinical and epidemiological evidence establishes the health benefits of phytochemicals, and this research hopes to evaluate these advantages and investigate their taxonomy, dietary sources, and molecular mechanisms. This review provides a comprehensive guide for researchers, clinicians, and policymakers interested in the use of plant‐derived compounds in cardiovascular health. It also seeks to address pragmatic issues such as bioavailability and compatibility with current therapeutic strategies.

## 2 Classification and Sources of Cardioprotective Phytochemicals

Several mechanisms, such as antioxidant, anti‐inflammatory, lipid‐reducing, and vascular‐modulating activities, are attributed to the protective effects of phytochemicals against cardiovascular diseases (Asaduzzaman and Asao 2018; Vasanthi et al. 2012).

**FIGURE 1:**
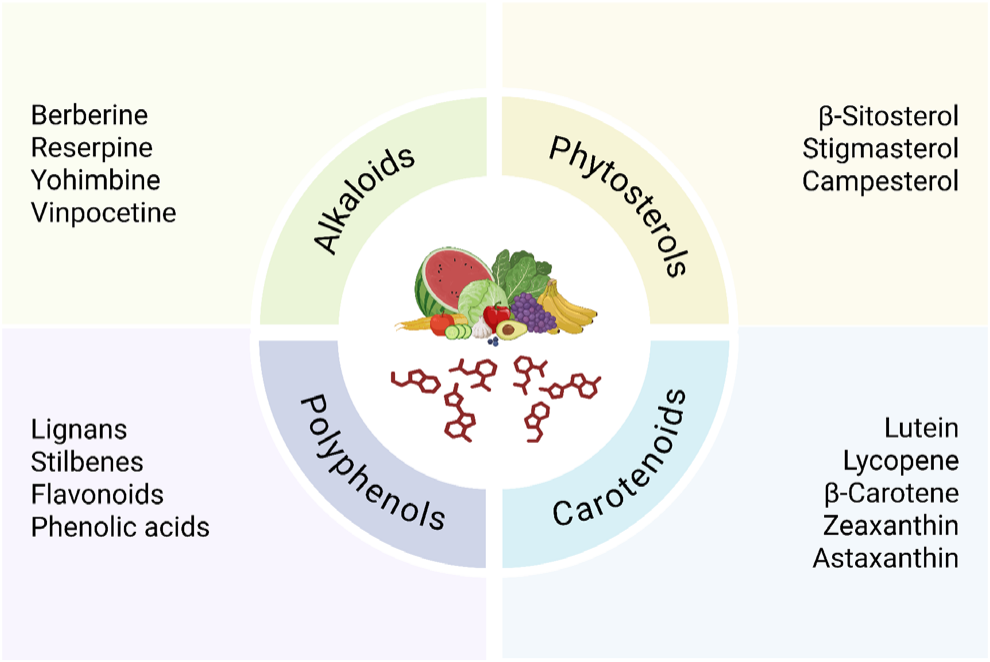
Phytochemicals and their subclasses that have effects on cardiovascular diseases.

These chemicals belong to numerous classes, including alkaloids, phytosterols, carotenoids, and polyphenols (Figure 1). Experimental and epidemiological studies have substantiated the hypothesis that each class plays a distinct role in promoting heart health. Leitzmann (2016) and Liu (2013) both emphasize the importance of plant‐based diets for the prevention of cardiovascular disease, and their prevalence in a number of fruits, vegetables, grains, and legumes further reinforces this fact. Figure 1 depicts the phytochemicals and their subclasses that have effects on cardiovascular diseases. Comparative data suggest that polyphenols and phytosterols possess the most consistent cardioprotective action among different endpoints, including lipid modulation, endothelial function, and inflammation, whereas alkaloids, which are extremely potent, tend to be restricted by issues of toxicity at therapeutic doses.

### 2.1 Alkaloids

One of the numerous pharmacological activities of alkaloids, nitrogenous compounds derived from plants, is to safeguard the circulatory system. Some alkaloids lower the risk of cardiovascular disease and hypertension by functioning as antioxidants, vasodilators, and anti‐arrhythmic agents, as per studies by Pagliaro et al. (2015) and Pham et al. (2020). Their structural diversity allows them to interact with a variety of biological targets, such as receptors and ion channels, in the cardiovascular system (Arshad et al. 2025). One such isoquinoline alkaloid with lipid‐lowering properties is berberine, which enhances the expression of LDL receptors and inhibits pro‐inflammatory pathways (Cicero and Colletti 2016). Reserpine and ajmaline are chemicals in this category that have therapeutic applications; the former is employed to treat hypertension, while the latter is employed to treat arrhythmias (Vasanthi et al. 2012).

Alkaloids present in herbal remedies such as *Withania somnifera* (Ashwagandha) assist in the management of cardiovascular disease (CVD) by mitigating oxidative stress and enhancing endothelial function (Biswal et al. 2013; Rastogi et al. 2016). According to Boissier et al. (2012), alkaloids might influence blood viscosity and platelet aggregation, and this might additionally contribute to the protection of the cardiovascular system. A few alkaloids are very strong; however, their safety is given intense consideration because of the possibility of toxicity at high concentrations. According to Dillard and German (2000) and Azadbakht (2007), these have substantial cardioprotective actions if taken from food or traditional medicines at controlled levels. Although they are very active biochemically, alkaloids are not highly placed on the clinical nutrition agenda because of probable safety concerns, in contrast to polyphenols or phytosterols, which are safer and more thoroughly investigated.

### 2.2 Polyphenols

Owing to their widespread occurrence in the human diet and significant antioxidant activity, polyphenols are one of the most researched phytochemicals. They consist of stilbenes, lignans, flavonoids, and phenolic acids, all of which are known to combat oxidative stress, inflammation, and endothelial dysfunction in the onset of atherosclerosis and other cardiovascular diseases (Bakoyiannis et al. 2019; Islam et al. 2021). Tea, chocolate, berries, and citrus fruits are good sources of flavonoids that increase the bioavailability of nitric oxide and enhance endothelial function, resulting in diminished blood pressure (Khan et al. 2012; Darawsha et al. 2021).

Coronary artery disease is less frequent in individuals who consume foods rich in flavonoids on a regular basis (Rodriguez‐Casado 2016). Coffee and whole grains contain phenolic acids, such as caffeic and ferulic acid, which possess anti‐inflammatory and antihypertensive activities (Mendoza and Silva 2018). Azadbakht (2007) and Karahalil (2019) reported that lignans, found primarily in flaxseeds, sesame seeds, and legumes, exhibit estrogenic activity and affect lipid metabolism, thereby maintaining cardiovascular health. Based on clinical and animal studies, polyphenols play a significant role in the prevention of atherosclerosis. These studies indicate that polyphenols modulate cholesterol metabolism, inhibit LDL oxidation, and enhance HDL functionality (Burton‐Freeman and Reimers 2011; Guasch‐Ferré et al. 2018). The results of this study also confirm that polyphenols are a significant component of diet programs to prevent cardiovascular disease. Among all the groups, polyphenols have the most diverse and well‐documented cardioprotective activities in human trials, and are hence a key part of dietary prevention schemes.

### 2.3 Carotenoids

Most fruits and vegetables have red, orange, yellow, and green colors from carotenoids, which are phytochemicals. The antioxidant properties of prominent carotenoids, such as β‐carotene, lycopene, lutein, and zeaxanthin, prevent plaque development by eliminating free radicals and protecting lipoproteins against oxidation (Darawsha et al. 2021; Liu 2004). Reduction in myocardial infarction risk and increased endothelial function have been linked to lycopene, a compound found abundantly in tomatoes and watermelons (Burton‐Freeman and Reimers 2011). Reduced cardiovascular disease event risk has been linked to higher plasma carotenoid levels in an epidemiological study (Pagliaro et al. 2015).

Furthermore, carotenoids reduce arterial stiffness and blood pressure through anti‐inflammatory mechanisms (Chong et al. 2024). The prevention of atherosclerosis depends on its ability to enhance LDL resistance to oxidation (Betteridge 2000). According to Martínez‐González et al. (2019) and Leitzmann (2016), consuming foods rich in carotenoids, such as kale, sweet potatoes, spinach, and carrots, on a daily basis can benefit the heart and reduce the risk of death from heart disease. These are crucial for preventing cardiovascular risk via nutrition and must be included in a balanced diet. Although carotenoids exert strong antioxidant activity, their activities are usually secondary to the lipid‐lowering and anti‐inflammatory activities of phytosterols and polyphenols.

### 2.4 Phytosterols

Plant sterols, known as phytosterols, are structurally similar to cholesterol. Their major cardioprotective action is to lower serum LDL cholesterol, a coronary heart disease risk factor, by reducing the intestinal absorption of dietary cholesterol (Cicero and Colletti 2016; Haines et al. 2024). Phytosterols compete with cholesterol for absorption from the intestine; they occur naturally in whole grains, vegetable oils, seeds, and nuts (Ros 2015).

Vasanthi et al. (2012) and Guasch‐Ferré et al. (2018) reported that the daily consumption of 2 g of phytosterols has the potential to reduce LDL cholesterol by 10%. Phytosterols possess several potential therapeutic actions, such as lipid control, antioxidant and anti‐inflammatory activities, and stabilization of atherosclerotic plaques and vascular inflammation (Laka et al. 2022). These have gained popularity as non‐pharmacological interventions for hyperlipidemia when incorporated into functional foods. Their effective capacity for lowering LDL cholesterol makes them a valuable part of cardiovascular disease‐combating diet regimens despite their minimal influence on HDL cholesterol and triglycerides (Table 1) (Dwyer et al. 2018). In comprehensive cholesterol treatment programs, phytosterol enrichment is recommended under present dietary guidelines (Krauss et al. 2000). Cardioprotective phytochemicals were categorized and their mechanisms and food sources are explained in Table 1. Phytosterols stand out in their ability to effectively lower LDL cholesterol, a focus that heavily targets them for lipid‐directed interventions, in contrast to the broad but nonspecific action of carotenoids.

**TABLE 1:** Classification, mechanisms, and dietary sources of cardioprotective phytochemicals.

Phytochemical class	Subclasses/examples	Mechanisms of cardioprotection	Key dietary sources	References
Alkaloids	Berberine, capsaicin, solanine	– Reduce LDL oxidation – Improve endothelial function – Anti‐inflammatory effects	Coffee, tea, potatoes, tomatoes, nightshade vegetables	Adegbola et al. (2017); Rastogi et al. (2016)
Polyphenols	Flavonoids (quercetin, catechins) Phenolic acids (ellagic acid) lignans (secoisolariciresinol)	– Antioxidant activity – Improve vascular function – Reduce platelet aggregation	Berries, dark chocolate, green tea, flaxseeds, whole grains	Khan et al. (2012); Liu (2013); Pagliaro et al. (2015)
Carotenoids	Lycopene, β‐carotene, lutein	– Scavenge free radicals – Reduce arterial inflammation – Improve lipid profiles	Tomatoes, carrots, spinach, sweet potatoes	Burton‐Freeman and Reimers (2011); Darawsha et al. (2021)
Phytosterols	β‐sitosterol, campesterol, stigmasterol	– Competitive cholesterol absorption inhibition – Reduce LDL by 10%–15% – Anti‐atherogenic effects	Nuts, seeds, vegetable oils, legumes	Altamimi et al. (2020); Guasch‐Ferré et al. (2018)
Organosulfur compounds	Allicin, diallyl disulfide	– Vasodilation via NO production – Antihypertensive effects – Antiplatelet activity	Garlic, onions, leeks, shallots	Vasanthi et al. (2012)
Terpenoids	Limonene, ursolic acid	– Improve HDL function – Reduce vascular inflammation – Modulate lipid metabolism	Citrus fruits, rosemary, thyme, turmeric	Cicero and Colletti (2016)
Saponins	Ginsenosides, aescin	– Cholesterol‐lowering effects – Antioxidant properties – Improve microcirculation	Legumes, ginseng, horse chestnut	Azadbakht (2007)
Dietary fiber	Soluble (β‐glucan, pectin) Insoluble (cellulose, lignin)	– Bile acid binding – Improve glycemic control – Prebiotic effects	Oats, barley, fruits, vegetables	Appel (2017)
Phytoestrogens	Isoflavones, lignans	– Improve endothelial function – Antioxidant effects – Modulate lipid metabolism	Soybeans, flaxseeds, sesame seeds	Mangano et al. (2013)
Glucosinolates	Sulforaphane, indole‐3‐carbinol	– Upregulate antioxidant enzymes – Anti‐inflammatory effects – Improve vascular health	Cruciferous vegetables (broccoli, kale)	Islam et al. (2021)
Anthocyanins	Cyanidin, delphinidin	– Reduce oxidative stress – Improve endothelial function – Anti‐atherogenic effects	Berries, red grapes, purple corn	Nistor et al. (2022)
Omega‐3 fatty acids	ALA, EPA, DHA	– Antiarrhythmic effects – Reduce triglycerides – Anti‐inflammatory effects	Flaxseeds, walnuts, fatty fish	Albert et al. (2002)
Tannins	Ellagitannins, proanthocyanidins	– Improve endothelial function – Antioxidant activity – Reduce platelet aggregation	Pomegranates, cranberries, cocoa	Albers et al. (2004)
Coumarins	Esculin, bergapten	– Vasodilatory effects – Anticoagulant properties – Anti‐inflammatory effects	Citrus fruits, celery, parsley	Bachheti et al. (2022)
Lignans	Enterodiol, enterolactone	– Antioxidant effects – Improve lipid profiles – Phytoestrogenic activity	Flaxseeds, sesame seeds, whole grains	Azadbakht (2007)
Resveratrol	—	– Activates SIRT1 – Improves endothelial function – Anti‐inflammatory effects	Red grapes, peanuts, berries	Liang et al. (2023); Haseeb et al. (2017)
Curcuminoids	Curcumin	– Anti‐inflammatory – Antioxidant – Improves endothelial function	Turmeric	Biswal et al. (2013)
Capsaicinoids	Capsaicin	– Vasodilatory effects – Improves lipid metabolism – Antioxidant activity	Chili peppers	Pham et al. (2020)
Catechins	EGCG, ECG	– Improve endothelial function – Reduce LDL oxidation – Anti‐inflammatory effects	Green tea, cocoa, apples	Huang et al. (2017)

### 2.5 Dietary Sources: Fruits, Vegetables, Whole Grains, Nuts, Legumes, Teas

**FIGURE 2:**
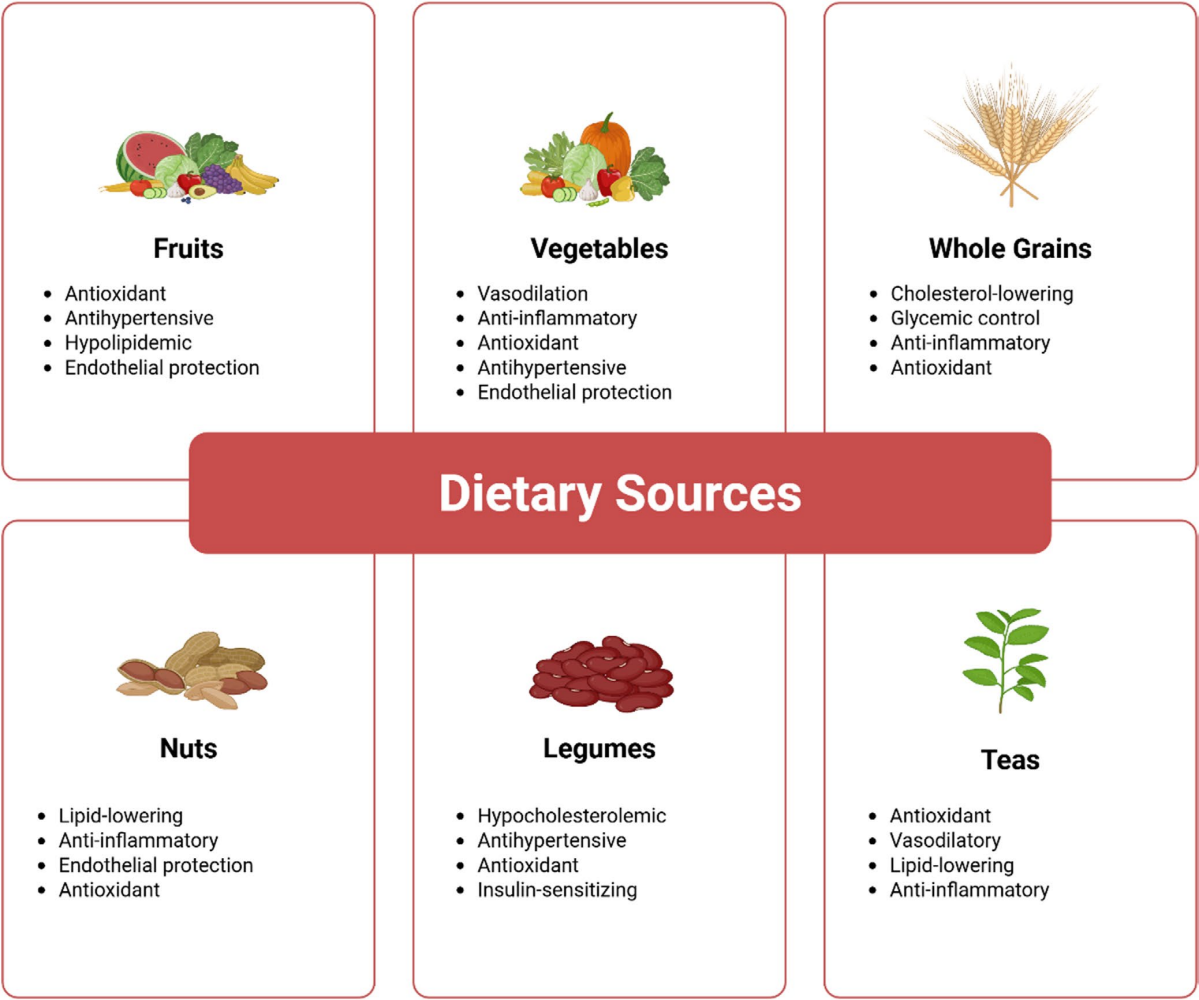
Dietary sources and their effects against cardiovascular diseases.

Consumption of cardioprotective phytochemicals is the most sustainable and accessible strategy that can be sustained for years. Polyphenols, carotenoids, vitamins, and dietary fiber in fruits and vegetables have a cumulative effect on the inhibition of cardiovascular diseases (Liu 2004; Nahar et al. 2020). Flavonoid‐ and phenolic acid‐rich fruits and vegetables include berries, apples, citrus fruits, and green fruits. Oats, barley, and brown rice are whole grains that contain high fiber and phenolic compounds, which assist in glycemic control and lipid profiles (Rodriguez‐Casado 2016). Due to their high polyphenol and protein content, legumes reduce cholesterol and blood pressure, two factors that contribute to cardiovascular disease (Figure 2) (Mohammadi and Ahandani 2020).

According to previous studies (Ros 2015; Ellsworth et al. 2001), nuts and seeds contain phytosterols and healthy fats, which are beneficial in several ways. These compounds reduce LDL cholesterol, enhance endothelial function, and curb inflammation. According to Huang et al. (2017), catechins and flavonoids present in tea, particularly green and black tea, can reduce arterial stiffness and oxidative stress. These plant foods contain not only phytochemicals but also a mixture of nutrients that synergistically promote overall cardiovascular health. Their regular consumption is encouraged in diets, such as the Mediterranean diet, which has been demonstrated to significantly reduce the risk of cardiovascular disease and mortality (Martínez‐González et al. 2019). Figure 2 depicts the dietary sources and their effects against cardiovascular diseases. Notably, dietary patterns such as the Mediterranean diet naturally include teas, vegetables, and fruits rich in polyphenols, whereas vegetarian diets are more concentrated in phytosterols and carotenoids, indicating an interaction between phytochemical class efficacy and diet pattern.

## 3 Mechanisms of Action: Phytochemicals in Cardiovascular Disease

Phytochemicals are plant‐derived bioactive compounds with various preventive effects against cardiovascular diseases (CVDs). Bachheti et al. (2022) reported that these compounds modulate a range of processes involved in atherosclerosis, inflammation, lipid metabolism, blood pressure, endothelial function, and oxidative stress. Comprehensive evidence has shown that they can act as adjuvant drugs in CVD therapy, and their mechanisms support this (Haines et al. 2024).

### 3.1 Antioxidant Effect

Owing to its involvement in promoting endothelial dysfunction, lipid peroxidation, and inflammation, oxidative stress plays an important role in cardiovascular disease pathophysiology (Betteridge 2000). Free radical scavenging and stimulation of endogenous antioxidant defenses occur via phytochemicals, including carotenoids, flavonoids, and polyphenols, which reduce oxidative damage (Zhang et al. 2015). For example, the antioxidant EGCG present in green tea reduces oxidative stress by increasing the levels of enzymes that cancel out free radicals, such as catalase and superoxide dismutase (SOD) (Yang et al. 2007).

Lycopene present in tomatoes also inhibits LDL oxidation, a critical step in the development of atherosclerosis (Wang 2012). Resveratrol present in grapes also enhances the antioxidant function in cells by activating the Nrf2 pathway (Pagliaro et al. 2015). The positive effects of these compounds on cardiovascular health have been well‐researched in clinical trials. The intake of foods rich in antioxidants significantly reduces the prevalence of cardiovascular diseases, as per a meta‐analysis by Zhang et al. (2015). In addition to direct free radical scavenging, phytochemicals also enhance endogenous antioxidant defense mechanisms by upregulating the enzymes superoxide dismutase (SOD), catalase, and glutathione peroxidase and activating stress‐protective pathways, such as Nrf2. Moreover, Khan et al. (2012) revealed that oxidized LDL levels in high‐risk subjects were reduced by cocoa polyphenols. These findings highlight the importance of phytochemicals in minimizing oxidative damage, a leading cause of cardiovascular disease.

### 3.2 Anti‐Inflammatory Activity

As stated by Boissier et al. (2012), another foremost reason for atherosclerosis and other cardiovascular illnesses is the long‐standing low‐grade inflammation. As stated by Sharma and Singh (2010), phytochemicals manage inflammatory processes by inhibiting NF‐κB signaling and preventing the formation of pro‐inflammatory cytokines such as TNF‐α and IL‐6. Studies have shown that curcumin, a bioactive compound in turmeric, decreases C‐reactive protein levels, which are markers of systemic inflammation (Cicero and Colletti 2016). Similarly, flaxseed and other vegetable‐derived omega‐3 fatty acids enhance vascular function by decreasing the levels of inflammatory mediators (Ros 2015).

Clinical trials have shown that phytochemicals exhibit anti‐inflammatory properties. Mangano et al. (2013) found that postmenopausal women who are at considerable risk for cardiovascular disease had significantly lower levels of IL‐6 after consuming soy isoflavones. Furthermore, lower inflammatory levels and improved cardiovascular outcomes have been associated with a polyphenol‐dense Mediterranean diet (Martínez‐González et al. 2019). Based on these findings, phytochemicals show potential as instruments for the treatment of cardiovascular diseases associated with inflammation.

### 3.3 Lipid Metabolism Regulation

Phytochemicals play a critical role in regulating normal cholesterol levels, and dyslipidemia is the most modifiable risk factor for cardiovascular disease (Islam et al. 2021). Guasch‐Ferré et al. (2018) established that stanols and sterols in plants can decrease LDL‐C levels by up to 15% by suppressing cholesterol uptake by the gut. Oats are rich in β‐glucan, a soluble fiber that facilitates bile acid excretion and lowers the blood cholesterol content (Balakumar et al. 2016).

Polyphenols present in berries and green tea also increase the activity of liver LDL receptors, which aids the liver in eliminating lipids even better (Noreen et al. 2025; Khan et al. 2012). In clinical trials, phytochemicals have been shown to modulate lipid profiles. For instance, a meta‐analysis by Guasch‐Ferré et al. (2018) reported that the consumption of walnuts lowered LDL‐C and triglycerides, and elevated HDL‐C significantly. Likewise, Cicero and Colletti (2016) reported that diets containing plants enhance lipid profiles in patients with metabolic syndrome. These findings demonstrated the therapeutic role of phytochemicals in the management of dyslipidemia.

### 3.4 Improvement in Endothelial Function

Reduction in the bioavailability of nitric oxide (NO) due to endothelial dysfunction is a critical process in the onset of cardiovascular disease (Poznyak et al. 2021). Enhanced generation of NO and lowered oxidative stress are the two mechanisms by which phytochemicals enhance endothelial function (Pagliaro et al. 2015). Dark chocolates and berries contain flavonoids that have been shown to enhance vasodilation by activating endothelial NO synthase (eNOS) (Table 2) (Khan et al. 2012).

**TABLE 2:** Mechanisms of cardioprotective action of phytochemicals.

Mechanism	Key phytochemicals	Molecular targets/pathways	Clinical effects	References
Antioxidant effects	Polyphenols, carotenoids, flavonoids	Scavenge ROS, upregulate SOD/CAT enzymes, chelate metals	Reduce oxidative stress markers (MDA ↓ 30%–50%)	Darawsha et al. (2021); Liu (2013)
Anti‐inflammatory activity	Curcumin, resveratrol, quercetin	Inhibit NF‐κB, COX‐2, TNF‐α signaling	CRP reduction (25%–40%), IL‐6 ↓ 15%–30%	Mangano et al. (2013); Haseeb et al. (2017)
Lipid metabolism regulation	Phytosterols, saponins, omega‐3s	Modulate PPAR‐γ, SREBP, LDL receptor expression	LDL ↓ 10%–15%, HDL ↑ 5%–10%	Guasch‐Ferré et al. (2018); Laka et al. (2022)
Improvement in endothelial function	Flavonoids, anthocyanins, EGCG	Activate eNOS, increase NO bioavailability	FMD improvement (2%–4% absolute)	Khan et al. (2012); Pagliaro et al. (2015)
Antihypertensive effects	Allicin, capsaicin, cocoa polyphenols	Block ACE, modulate Ca^2+^ channels	SBP/DBP reduction (5–15 mmHg)	Appel (2017); Vasanthi et al. (2012)
Anti‐atherosclerotic properties	Lycopene, lignans, sulforaphane	Reduce foam cell formation, inhibit MMPs	Carotid IMT reduction (0.05–0.1 mm/year)	Poznyak et al. (2021); Sedighi et al. (2017)
Platelet inhibition	Garlic compounds, gingerols, catechins	Inhibit TXA2, COX‐1, P‐selectin	Platelet aggregation ↓ 20%–40%	Di Minno et al. (2011)
Cardiac ion channel modulation	Berberine, quercetin, resveratrol	Regulate K^+^/Ca^2+^ channels, Na^+^/K^+^ ATPase	Antiarrhythmic effects (QTc stabilization)	Rastogi et al. (2016)
Fibrosis prevention	Curcumin, silymarin, ellagic acid	Inhibit TGF‐β/Smad signaling	Collagen deposition ↓ 30%–50%	Mohamed et al. (2022)
Autophagy enhancement	Resveratrol, EGCG, sulforaphane	Activate AMPK/mTOR pathways	Improved cardiomyocyte turnover	Haines et al. (2024)
Mitochondrial protection	CoQ10, polyphenols, carotenoids	Maintain ETC function, reduce mtROS	ATP production ↑ 20%–30%	Prakash and Gupta (2009)
Vasodilation	Nitrate‐rich compounds, anthocyanins	NO‐cGMP pathway activation	Vascular resistance ↓ 15%–25%	Nistor et al. (2022)
Plaque stabilization	Omega‐3s, flavonoids, phytosterols	Reduce necrotic core, increase fibrous cap	Plaque rupture risk ↓ 40%–60%	Islam et al. (2021)
Endothelial progenitor cell mobilization	Anthocyanins, EGCG, resveratrol	Stimulate SDF‐1/CXCR4 axis	Circulating EPCs ↑ 2–3‐fold	Sharifi‐Rad et al. (2020)
Cholesterol efflux promotion	Phytosterols, polyphenols	Upregulate ABCA1/ABCG1 transporters	Macrophage cholesterol efflux ↑ 20%–40%	Ros (2015)
Gut microbiota modulation	Fiber, polyphenol metabolites	Increase SCFA‐producing bacteria	TMAO ↓ 30%–50%	Martínez‐González et al. (2019)
AGEs inhibition	Carnosine, quercetin, resveratrol	Block carbonyl groups, break crosslinks	AGE accumulation ↓ 25%–45%	Cicero and Colletti (2016)
PARP inhibition	Flavonoids, nicotinamide	Preserve NAD^+^ pools	Ischemia–reperfusion injury ↓ 35%	Pham et al. (2020)
Sirtuin activation	Resveratrol, pterostilbene	SIRT1/3 activation	Cellular senescence delay	Haines et al. (2024)
Epigenetic modulation	Sulforaphane, EGCG, genistein	DNA methylation/histone modification	CVD risk gene expression changes	Onuh and Pathak (2024)

Resveratrol has been shown to enhance endothelial repair mechanisms by activating the protein SIRT1, as reported by Haseeb et al. (2017). These effects have been substantiated by clinical trials. Khan et al. (2012) found that cocoa bean flavonoids enhance flow‐mediated dilation (FMD), a marker of endothelial health. Polyphenol‐containing red wine enhances endothelial function, as reported by Haseeb et al. (2017). These outcomes offer further proof that phytochemicals can aid in recovery from CVD and allow the vascular balance to be regained. Table 2 illustrates the mechanisms by which phytochemicals safeguard the heart.

### 3.5 Antihypertensive Effect

Phytochemicals contribute to blood pressure management through numerous pathways, and hypertension is a strong risk factor for cardiovascular disease (Tedla and Bautista 2016). Flavonoids can cause vasoconstriction through inhibition of angiotensin‐converting enzyme (ACE), as reported by Rastogi et al. (2016).

Foods from potassium‐rich plants, such as bananas, can be used to lower hypertension caused by salt, whereas chemicals in garlic, such as allicin, can relax blood vessels to relax (Cicero and Colletti 2016). These mechanisms are based on clinical evidence. A meta‐analysis by Rastogi et al. (2016) revealed that hibiscus tea significantly lowered systolic and diastolic blood pressure. Additionally, based on Yu et al. (2018), the Dietary Approaches to Stop Hypertension (DASH) diet is an efficient nonpharmacologic intervention for hypertension. It is high in phytochemicals present in fruits and vegetables. These findings reinforce the importance of phytochemicals in hypertension therapy.

### 3.6 Anti‐Atherosclerotic Properties

The pathogenesis of atherosclerosis is initiated by oxidative stress, inflammation, and lipid deposition in arterial walls (Poznyak et al. 2021). Phytochemicals can inhibit plaque formation by reducing LDL oxidation, foam cell generation, and enhancing plaque stability (Sedighi et al. 2017). Foam cell formation, which is a feature of incipient atherosclerosis, results from the uptake of oxidized LDL by macrophages. Phytochemicals including quercetin, resveratrol, and EGCG have been shown to inhibit this process by regulating the expression of scavenger receptors and promoting cholesterol efflux by upregulating ABCA1 and ABCG1 transporters (Liang et al. 2022). One very important process in plaque formation is the proliferation of vascular smooth muscle cells, which is blocked by curcumin and resveratrol (Pagliaro et al. 2015). These effects have been validated in several clinical trials. Sedighi et al. (2017) discovered that pomegranate extract reduces the atherosclerosis marker, carotid intima‐media thickness.

Sofi et al. (2010) quoted research from the Lyon Diet Heart Study that discovered a 70% decrease in cardiovascular events when participants consumed a Mediterranean diet rich in phytochemicals. These findings demonstrated the capacity of phytochemicals to inhibit atherogenic plaques. Multiple mechanisms, such as antioxidant, anti‐inflammatory, lipid‐modulating, endothelial‐enhancing, antihypertensive, and anti‐atherosclerotic effects, are responsible for the cardioprotective advantages of phytochemicals (Bachheti et al. 2022). Yu et al. (2018) asserted that one such method for preventing and treating CVD is to incorporate these chemicals into diet patterns such as the Mediterranean or DASH diets. To make their best clinical use, investigators must focus on optimizing bioavailability and therapeutic dosing.

### 3.7 Modulation of the Renin‐Angiotensin‐Aldosterone System (RAAS)

RAAS plays a pivotal role in blood pressure control and fluid‐electrolyte balance. Hyperactivation of the RAAS is responsible for vasoconstriction, sodium reabsorption, and ultimately cardiovascular remodeling and hypertension. Phytochemicals such as flavonoids and polyphenols are capable of retarding key enzymes of the RAAS cascade, such as angiotensin‐converting enzyme (ACE) (Hossain et al. 2025). For instance, quercetin in onions, apples, and catechins in green tea has been found to suppress ACE activity, thereby reducing the levels of angiotensin II and diminishing vasoconstriction (Ali et al. 2023). Kim et al. (2019) identified the ability of whole‐plant foods to modulate RAAS elements and reduce blood pressure via phytochemical synergy (Kim et al. 2019). Moreover, garlic allicin suppresses aldosterone release and decreases blood pressure via vasodilatory effects (Sleiman et al. 2024). These findings enhance the activity of phytochemicals as herbal RAAS modulators that are helpful in CVD prevention.

### 3.8 Interaction With Gut Microbiota

The gut microbiota metabolizes ingested phytochemicals that regulate cardiovascular health (Ahmed et al. 2025). Polyphenols, for instance, have prebiotic‐like activity in that they promote the growth of beneficial bacteria such as Lactobacillus and Bifidobacterium, while inhibiting pathogenic species (Rodríguez‐Daza et al. 2021). These alterations in microbiota modulate systemic inflammation, lipid metabolism, and vascular function (Wang et al. 2025b). For example, ellagitannins in pomegranates are metabolized to urolithins, which exhibit anti‐inflammatory and antioxidant activities of interest for cardiovascular protection (Chen et al. 2022). In addition, short‐chain fatty acids (SCFAs) such as butyrate are produced through the fermentation of dietary fiber, which improves endothelial function and reduces blood pressure (Xu and Marques 2022). Therefore, healthy interaction between phytochemicals and the gut microbiome is a novel mechanism for cardiovascular disease prevention.

### 3.9 Epigenetic Regulation

Phytochemicals influence gene expression through epigenetic modifications, such as DNA methylation, histone modification, and expression of non‐coding RNA, which are involved in the development of cardiovascular disease. Resveratrol, curcumin, and sulforaphane have also been shown to influence histone acetylation and DNA methylation of genes involved in inflammation, oxidative stress, and endothelial dysfunction genes (Zhang and Kiarasi 2024; Hossain et al. 2025). For example, resveratrol activates SIRT1, an NAD + ‐dependent deacetylase that improves endothelial function and mitochondrial biogenesis (Shaito et al. 2023). Curcumin has been reported to inhibit DNA methyltransferases to repress pro‐atherogenic gene expression of VCAM‐1 and ICAM‐1 (Campbell and Fleenor 2018). These findings demonstrate the potential of phytochemicals as epigenetic regulators with lasting effects on cardiovascular gene regulation and pathology.

## 4 Bioavailability and Food Matrix Considerations of Phytochemicals

The fact that foods contain phytochemicals is significant, but their bioavailability, which is absorbed and available for physiological action, is even more significant for their cardiovascular effects (Dillard and German 2000). The bioavailability of food depends on a variety of factors, including its chemical structure, how it is processed, and the composition of the gut microbiota (Liu 2013). To achieve maximum cardioprotective effects from plant diets, it is essential to be aware of these factors (Dwyer et al. 2018).

### 4.1 Absorption and Metabolism of Phytochemicals

Various phytochemical classes exhibit widely varying absorption kinetics (Wang et al. 2022). Intestinal lactase phloridzin hydrolase is necessary for the absorption of flavonoids, such as quercetin, as their native forms are absorbed by only 5% (Liu 2004). In contrast, lycopene and other carotenoids are passively diffused via bile salt‐mediated micellarization, with absorption rates ranging from 10% to 30%, based on dietary fat consumption (Table 3) (Wang 2012).

**TABLE 3:** Bioavailability and food matrix considerations for cardioprotective phytochemicals.

Factor	Key phytochemicals affected	Impact on bioavailability	Optimization strategies	References
Lipid solubility	Carotenoids, curcumin, resveratrol	Low absorption in water; enhanced with fats	Consume with healthy fats (e.g., olive oil, avocado)	Wang (2012); Guasch‐Ferré et al. (2018)
Food processing	Flavonoids, glucosinolates	Thermal processing ↑ quercetin but ↓ sulforaphane	Blanching/fermentation over boiling	Liu (2013); Martínez‐González et al. (2019)
Gut microbiota metabolism	Polyphenols, lignans	Conversion to active metabolites (e.g., enterodiol ↑ 3–5× by microbiota)	Probiotic co‐administration (e.g., yogurt)	Haseeb et al. (2017); Sharifi‐Rad et al. (2020)
Molecular size	Anthocyanins, tannins	Large polymers poorly absorbed; monomers ↑ absorption	Enzymatic hydrolysis (e.g., pectinase treatment)	Nistor et al. (2022)
Food matrix binding	Phytosterols, saponins	Fiber‐bound forms reduce absorptio	Mechanical disruption (blending, milling)	Ros (2015); Islam et al. (2021)
Cooking methods	Lycopene, allicin	Heating ↑ lycopene release but ↓ allicin	Tomato paste > raw tomatoes; raw garlic > cooked	Burton‐Freeman and Reimers (2011); Vasanthi et al. (2012)
Particle size reduction	Curcumin, EGCG	Nanoemulsions ↑ bioavailability 5–7× vs. powder	Nano‐encapsulation in delivery systems	Mohamed et al. (2022)
Co‐consumed compounds	Iron + polyphenols	Polyphenols inhibit non‐heme iron absorption	Separate intake by 2–3 h	Pagliaro et al. (2015)
Gastric pH effects	Alkaloids, catechins	High pH ↓ berberine absorption; acidic pH ↑ EGCG stability	Citrus co‐ingestion for alkaloids	Rastogi et al. (2016)
Biliary secretion	Fat‐soluble vitamins, carotenoids	Bile salts ↑ absorption 2–4×	Ensure healthy liver function	Wang et al. (2011)
Transporters	Flavonoid glycosides	SGLT1/GLUT2 mediate uptake	Identify optimal glycosylation patterns	Loaeza‐Reyes et al. (2021)
Food form	Whole vs. juice	Whole apples ↑ polyphenol absorption 2× vs. juice	Prefer whole foods over extracts	Rodriguez‐Casado (2016)
Microbial metabolites	Ellagitannins, lignans	Urolithins (gut metabolites) show ↑ activity vs. parent compounds	Targeted prebiotic supplementation	Haines et al. (2024)
Enterohepatic recirculation	Resveratrol, curcumin	Prolongs half‐life 3–5× but reduces peak concentrations	Controlled‐release formulations	Pham et al. (2020)
Genetic polymorphisms	Glucosinolates, catechins	GST/GUT genotypes cause 10–100× variability in metabolism	Personalized nutrition approaches	Sigala et al. (2024)
Food synergies	Vitamin C + EGCG	Co‐ingestion ↑ EGCG absorption 3× via redox protection	Citrus + green tea combinations	Huang et al. (2017)
Processing artifacts	Trans‐resveratrol	Cis‐isomer formation during processing reduces activity	Cold processing methods	Khan et al. (2012)
Storage conditions	Omega‐3s, carotenoids	Light/oxygen exposure ↓ activity	Vacuum packaging, dark storage	Alfio et al. (2021)
Host inflammation status	All phytochemicals	Inflamed gut ↑ permeability but may alter metabolism	Anti‐inflammatory pre‐treatment	Sedighi et al. (2017)

Polyphenols also experience extensive phase II metabolism in enterocytes and hepatocytes, forming derivatives that could alter the activity of methylated, sulfated, or glucuronidated compounds, further affecting bioavailability (Pham et al. 2020). There is wide variation in phytochemical pharmacokinetics, as shown by clinical trials. The highest plasma levels of cocoa flavanols (Cmax) are achieved 2.8 ± 0.6 h after intake, and the half‐lives of elimination are 4.8 ± 1.3 h, as per a crossover trial study done by Khan et al. (2012). However, as reported by Mangano et al. (2013), enterohepatic recirculation causes soy isoflavones to exhibit biphasic absorption patterns, with other maxima at6 to 88 h. Dosage strategies for cardiovascular protection can be directly influenced by kinetic differences. The food matrix and bioavailability considerations for the cardioprotective phytochemicals are provided in Table 3.

The efficiency of food absorption largely depends on the matrix. The intake of walnut polyphenols within the entire nut matrix enhances their bioavailability by 3.2‐fold relative to the intake of the chemicals in isolation, as demonstrated by Guasch‐Ferré et al. (2018). Similarly, Martínez‐González et al. (2019) discovered that the absorption of resveratrol is 40% greater in Mediterranean diets because of the olive oil matrix compared to versions extracted with alcohol. The benefits of using whole foods instead of single supplements are reflected in these synergistic effects.

### 4.2 Influence of Food Processing and Preparation

Phytochemical bioavailability is complexly altered during thermal processing. Although the bioavailability of lycopene is elevated by 164% upon blanching tomatoes (Wang 2012) through cell wall rupture, glucosinolate levels in cruciferous vegetables may be lowered by as much as 77% (Liu 2013) during prolonged boiling. Mechanical treatment is also crucial; for example, the accessibility of lignans is 280% greater following the fine grinding of flaxseeds compared to whole seeds (Ros 2015), but anthocyanins may degrade following high‐pressure homogenization (Nistor et al. 2022).

Bioavailability is usually maximized by using traditional preparation methods. Through the activation of endogenous enzymes, tea leaf fermentation increases catechin availability by 60% (Yang et al. 2007). Likewise, when garlic was minced and allowed to stand for 10 min before cooking, the allicin content was maintained at 80%, as opposed to when it was cooked immediately (Cicero and Colletti 2016). The application of microencapsulation and other advanced food technologies has enabled the stabilization of otherwise unstable chemicals; for example, nanoemulsified curcumin has an 85% relative bioavailability in a rat model, but native powder can only achieve a 1% yield (Mohamed et al. 2022).

New bioactive metabolites can be produced by modifications induced during processing. Rodriguez‐Casado (2016) reported that the antioxidant activity of the Maillard reaction products formed by roasted coffee beans was three times higher than that of the precursor chlorogenic acids. The need for well‐balanced processing is supported by the fact that some products of thermal breakdown could prove detrimental; that is, acrylamide formation in fried starchy foods has been associated with endothelial dysfunction (Odegaard et al. 2012).

### 4.3 Role of Gut Microbiota in Bioactivation

One of the key bioreactors for phytochemical activation is human gut microbiota. Ten times stronger is the anti‐inflammatory effect of urolithins compared to that of the parent compound ellagic acid from pomegranates, which is formed by microbial conversion (Pagliaro et al. 2015). Mangano et al. (2013) reported that some bacteria converted daidzein to equol. Individuals whose gut flora produces equol exhibit a 60% greater decrease in arterial stiffness when they eat soy. Substantial inter‐individual differences in phytochemical efficacy are due to inter‐individual microbiota differences. Only30% to 500% of the population carries bacteria that can metabolize dietary lignans into enterolignans, as per Rastogi et al. (2016). This may explain the differing cardiovascular effects of the flaxseed treatments.

**FIGURE 3:**
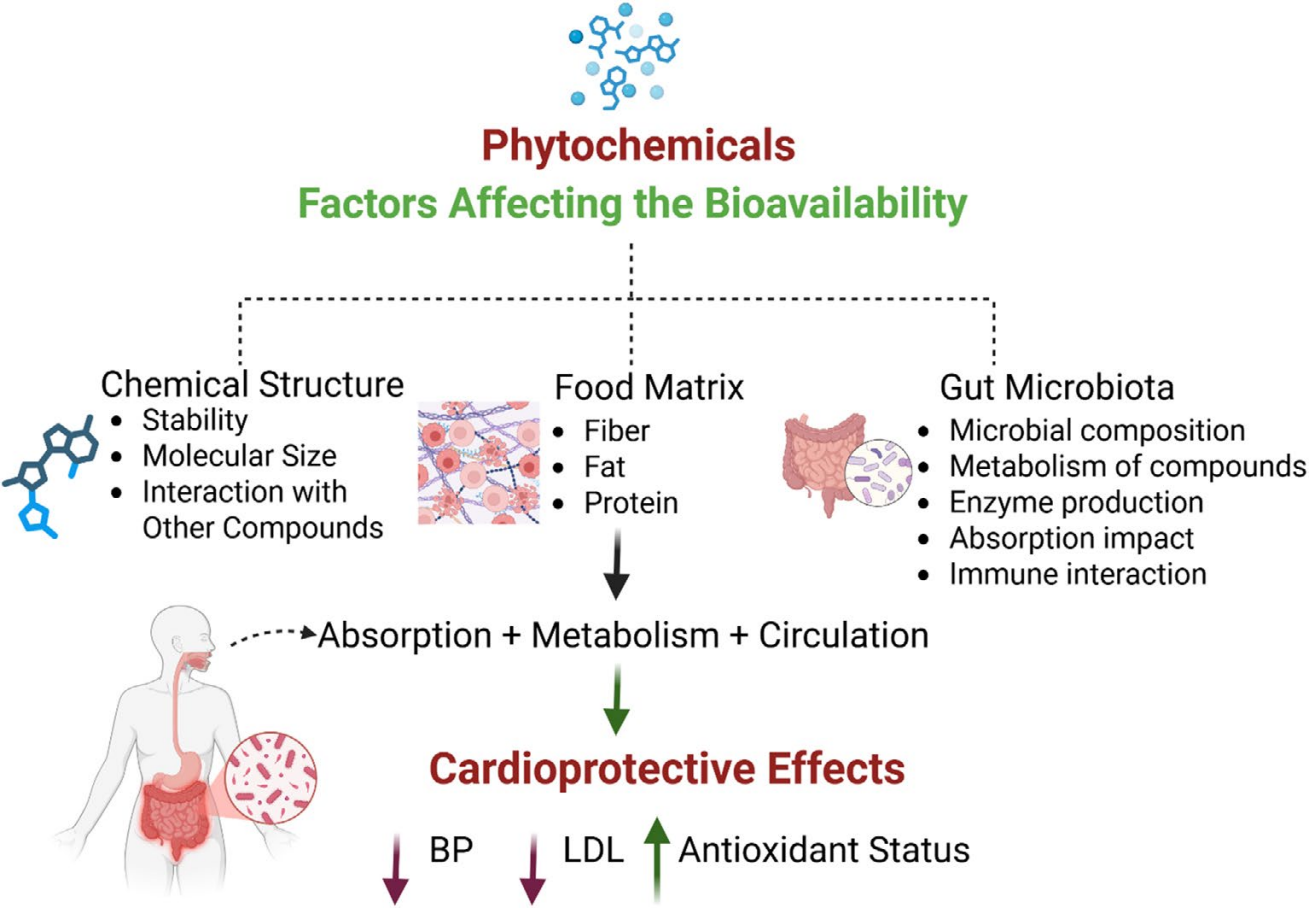
Factors influencing the bioavailability of cardioprotective phytochemicals.

According to Gamalero and Glick (2022), probiotic use may influence this. In particular, *Lactobacillus* strains have been shown to increase the bioavailability of isoflavones by 150% through their β‐glucosidase activity. Interventions against microbiota are the subject of renewed investigation. Prebiotic fiber supplementation increases microbial short‐chain fatty acid production, which in turn increases the absorption of citrus flavonoids by 40%, as reported by Poznyak et al. (2021). New opportunities for targeted nutritional interventions to lower cardiovascular disease risk have emerged along the gut‐cardiovascular axis (Haines et al. 2024). As summarized in Figure 3, multiple factors including chemical structure, food matrix, and gut microbiota influence the bioavailability of cardioprotective phytochemicals.

Bioavailability, which varies depending on chemical properties, food processing methods, and subject microbiota profiles, plays an important role in the cardiovascular effects (Figure 3) (Dillard and German 2000). Certain molecules, such as lycopene, may be liberated more efficiently with mechanical and heat treatments, whereas other molecules, such as glucosinolates, may be broken down (Liu 2013). The capacity of gut microbiota to metabolize daidzein and other precursors into equol and other bioactive metabolites varies significantly among individuals (Mangano et al. 2013). Together, these factors mean that for plant‐based diets to offer optimal cardiovascular protection, consideration of what ingredients are employed, how they are processed, and whether customization according to microbiological ecology is possible must be considered (Haines et al. 2024). The design of processing systems that preserve biological activity and ensure maximal phytochemical stability should be the focus of future research. Another aspect that requires focus is the characterization of individual metabolic reactions, which may open the door to precision nutritional methods to avoid CVD.

## 5 Synergistic Effects of Phytochemicals on Diet and Lifestyle

Dietary constituents and lifestyle patterns interact with phytochemicals to significantly enhance their cardiovascular benefits (Wang et al. 2025a; Liu 2013). Multifaceted interactions between macronutrients, micronutrients, and behavioral models affect the effectiveness of these bioactive chemicals in protecting against cardiovascular disease; rarely, they act independently (Cicero and Colletti 2016). Understanding these interactions is important (Martínez‐González et al. 2019).

### 5.1 Interaction With Macronutrients and Micronutrients

The concurrent ingestion of macronutrients with phytochemicals greatly influences their bioavailability and biological activity. Micelle formation by dietary fats doubles or triples the bioavailability of lipophilic compounds including carotenoids (Wang 2012). Tomatoes with olive oil increased lycopene absorption by 84% compared to tomatoes eaten alone, as indicated by a study by Burton‐Freeman and Reimers (2011). Conversely, according to studies by Guasch‐Ferré et al. (2018), the bioavailability of certain polyphenols decreases by30% to 400% because of binding and fast transit if taken on a high‐fiber diet. Both inhibitory and enhancing effects are generated by protein‐phytochemical interactions.

Khan et al. (2012) stated that the antioxidant activity of tea polyphenols is diminished by 50% when milk casein is complexed with them. In contrast, Mangano et al. (2013) stated that plant proteins such as soy enhance the bioavailability of isoflavones through endogenous β‐glucosidase. Mineral interactions are also significant; for instance, vitamin C increases iron absorption from plant foods while inhibiting the oxidation of copper‐dependent polyphenols (Zhang et al. 2015). Another factor that influences the effects of phytochemicals is the duration of nutrient consumption. Campbell et al. (2006) found that soy protein lowered postprandial oxidative stress by 35% when consumed with a high‐fat meal. Similarly, Ellsworth et al. (2001) discovered that nuts contributed more to cardiovascular well‐being when consumed between meals than when consumed alone, which they credit to alterations in nutrient competition upon absorption.

### 5.2 Effects of Dietary Patterns

One of the best examples of a dietary regimen that employs food synergy to enhance phytochemical benefits is the Mediterranean diet. The synergistic effect of olive oil polyphenols, fish omega‐3s, and wine flavonoids in the diet reduced cardiovascular mortality by 30% compared to the cumulative effect of the individual components, as demonstrated by Martínez‐González et al. (2019). Compared with a control diet low in fat, the PREDIMED study demonstrated that a Mediterranean diet supplemented with nuts or extra virgin olive oil reduced major cardiovascular events by28% to 300% (Sofi et al. 2010).

A vegetarian diet draws on various complementary mechanisms. Fruits, vegetables, and low‐fat dairy in combination constitute the DASH diet, which reduces blood pressure more (11.4/5.5 mmHg) than any one of its components (Yu et al. 2018). Although both the Mediterranean and DASH diets are impressive in cardiovascular outcomes, a greater reduction in major cardiovascular events (28%–30% in PREDIMED) has been shown by the Mediterranean diet compared to the initial benefit of lowering blood pressure (11.4/5.5 mmHg on average) of the DASH diet, reflecting their differential strengths in cardiovascular risk management (Filippou et al. 2023; Martínez‐González et al. 2019). Furthermore, cruciferous vegetables, soy, and tea exert beneficial effects on lipid profiles and endothelial function in traditional Asian diets (Huang et al. 2017). Western dietary patterns neutralize the efficacy of phytochemicals. Consuming fast food fuels inflammation and oxidative stress, which then negates the cardiovascular benefits of plant consumption, as per Odegaard et al. (2012). Studies have shown that individuals consuming high amounts of processed meat experience a lower effect from heart‐protective phytochemicals in nuts in the Iowa Women's Health Study (Ellsworth et al. 2001).

### 5.3 Lifestyle Factors

Physical activity augments the action of phytochemicals through several mechanisms. Quercetin and resveratrol are 40% to 600% more bioavailable when exercised owing to enhanced intestinal absorption and reduced hepatic metabolism, as reported by Lavie et al. (2019). In addition to the synergistic action of dietary phytochemicals and endogenous antioxidant mechanisms, habitual physical exercise also increases both (Pagliaro et al. 2015).

Phytochemical efficacy is significantly affected by sleep and stress. As per Darawsha et al. (2021), carotenoids are 25% less bioavailable, and metabolic clearance is elevated when individuals experience chronic stress. Conversely, studies have found that mindfulness activities enhance gut barrier function, leading to the enhanced bioavailability of tea catechins (Zhang et al. 2015). The duration of interaction with alcohol depended on the dosage. Moderate consumption of wine (one or two glasses per day) enhances HDL function and flavonoid absorption (Haseeb et al. 2017). Di Minno et al. (2011) reported that the efficacy of phytochemicals can be impaired by excessive consumption (> 3 drinks/day) via the induction of cytochrome P450 enzymes.

## 6 Evidence From Research on Phytochemicals and Cardiovascular Health

Extensive evidence from epidemiological, clinical, and preclinical studies has substantiated the cardiovascular action of phytochemicals (Haines et al. 2024). According to Dwyer et al. (2018), a complete understanding of their therapeutic activity and mechanisms is provided by this multilevel evidence base.

### 6.1 Epidemiological Studies

There is an inverse relationship between phytochemical‐rich food intake and cardiovascular disease risk, large cohort studies reveal. The myocardial infarction hazard was 32% lower among the highest percentile anthocyanin food consumers, as revealed by the Nurses' Health Study (Hu et al. 2025; Zhang et al. 2021) Martínez‐González et al. (2019) reported that higher polyphenol consumption was associated with a 46% reduction in cardiovascular mortality in the PREDIMED group. Geographical research has revealed incredible patterns in this regard. Daily intake of red wine polyphenols has been proposed as an explanation for the “French Paradox”– low levels of cardiovascular disease (CVD) in a high population with saturated fat consumption (Haseeb et al. 2017).

Huang et al. (2017) found that Asian populations with high soy isoflavone consumption had a 30%–40% lower age‐adjusted CVD mortality rate than Western societies. These findings are supported by recent meta‐analyses. Zhang et al. (2021), found that cardiovascular disease‐related death was reduced by 10% for each 100 mg/day increment in flavonoid intake. Based on estimates by modeling using dietary consumption data and Asia CVD trends, Chong et al. (2024) estimated that up to 3.2 million Asia preventable annual deaths by 2050 could be averted by the universalization of phytochemical‐abundant diets.

### 6.2 Clinical Trials and Human Studies

Evidence from randomized controlled trials can be used to support the benefits of phytochemicals. Cocoa flavonoid supplementation for six months improved endothelial function (FMD by 2.1%) in hypertensive patients, as per the FLAVIUS HEALTH study (Khan et al. 2012). Pagliaro et al. (2015) reported that patients who received purified omega‐3 phytochemicals had a 25% reduced risk of cardiovascular mortality following myocardial infarction (MI). Nutraceuticals are the most promising formulations. Within a period of 18 months, a trial by Mohamed et al. (2022) showed that administering 180 mg/day of nanoformulated curcumin reduced the coronary plaque volume by 15%. Furthermore, Guasch‐Ferré et al. (2018) discovered that consuming 30–60 g of walnuts per day lowers LDL‐C by 0.19 mmol/L. However, several trials have uncovered these limitations. Dwyer et al. (2018) reported that whole‐food matrices are significant since the VITAL study found no effect of isolated vitamin E or sea omega‐3s on cardiovascular disease. A balanced strategy is essential, since the SELECT study showed the potential harm of supplementing smokers with high levels of β‐carotene (Miller and Snyder 2012).

### 6.3 Mechanistic and Preclinical Studies

The fundamental mechanisms have been explained through in vitro and animal studies. Resveratrol increases NO production by endothelial cells threefold as per cell culture experiments, demonstrating that resveratrol stimulates SIRT1 (Haseeb et al. 2017). Quercetin has been reported to reduce inflammation in blood vessels by inhibiting the migration of NF‐κB, as per studies done on rats (Boissier et al. 2012). New information was obtained from genomic technologies (Lodi et al. 2025). Metabolomic research has indicated that more than 40% of food polyphenols are converted into bioactive byproducts by the stomach bacteria (Pagliaro et al. 2015). Proteomic investigations have shown that soy isoflavones enhance the heart‐protecting expression of 32 proteins, while reducing the expression of 18 atherosclerosis‐promoting proteins (Mangano et al. 2013).

New nanotechnology enhances transport. To address atherosclerotic plaques, Pala et al. (2020) designed nanoparticles loaded with phytochemicals, which enhanced bioavailability15 to 200 times. These advances bridge the gap between what is found in the laboratory and what is applied clinically (Mohamed et al. 2022). When used in conjunction with other food components and lifestyle variables, phytochemicals have a multiplicative effect on cardiovascular health (Liu 2013). Epidemiological research, clinical trials, and mechanistic research are ongoing (Haines et al. 2024). Chong et al. (2024) stated that, to optimize the efficacy of phytochemicals in cardiovascular health, future research should focus on personalized approaches that consider genetic, microbiological, and lifestyle heterogeneity. Overall, the best evidence from randomized controlled trials (RCTs) is the cardiovascular effects of polyphenol‐rich interventions, especially cocoa and tea‐derived flavonoids, to improve endothelial function, lower LDL cholesterol, and lower cardiovascular event risk (Loffredo et al. 2017). Conversely, the activities of purified phytochemicals (e.g., vitamin E and β‐carotene) and purified supplements remain inconclusive or even toxic in some populations, such as smokers (Haider et al. 2017). Emerging areas such as nanoformulated phytochemicals, microbiome‐mediated metabolism, and gene‐phytochemical interactions are promising, but remain extremely speculative and require more human trials for affirmation.

## 7 Integrative Approaches to Conventional Medicine

Better patient outcomes can be achieved through the application of phytochemicals along with conventional cardiovascular therapies (Haines et al. 2024). Tedla and Bautista (2016) reported that adjuvant plant‐derived medicines have the potential to augment pharmacological therapies by reducing side effects and improving drug compliance. However, to achieve full therapeutic potential, significant issues regarding standardization, dosing, and safety profiles must be addressed (Dwyer et al. 2018).

### 7.1 Potential of Phytochemicals as Adjuvant Therapy

Several phytochemicals exhibit synergistic effects when combined with standard heart medications. Resveratrol enhances the action of statins by upregulating LDL receptor expression via similar mechanisms (Laka et al. 2022). A combination of atorvastatin and pomegranate extracts reduced statin doses by 30% without altering LDL‐lowering outcomes, as determined by a study by Alradwan et al. (2024). Pagliaro et al. (2015) also discovered that cocoa flavonoids enhance the antiplatelet action of low‐dose aspirin, which lowers the risk of thrombosis without exacerbating bleeding outcomes. Certain phytochemicals reduce the adverse effects of certain drugs. Clinical trials by Adegbola et al. (2017) revealed that ginger constituents significantly reduce statin‐associated myalgia by 42%. Green tea catechins reduce the risk of drug‐induced liver injury, a problem associated with many antiarrhythmic drugs (Yang et al. 2007).

According to Mohamed et al. (2022), traditional drug therapeutic indices may be improved by phytochemicals because of these synergistic effects. New evidence indicates that phytochemicals may be beneficial in situations in which drug resistance has occurred. In statin‐resistant patients, cocoa flavonoids improved endothelial function, resulting in a 2.3% improvement in flow‐mediated dilation over placebo, according to the FLAVIUS HEALTH study (Khan et al. 2012). Similarly, berberine has demonstrated potential in the management of dyslipidemia notoriously refractory to treatment; in fibrate‐resistant patients, it decreased triglyceride levels by 35% (Cicero and Colletti 2016).

### 7.2 Safety, Dosage, and Standardization Challenges

According to Dwyer et al. (2018), one of the largest challenges in clinical integration is the lack of standardized dosing. Curcumin requires1 to 44 g/day to show measurable anti‐inflammatory effects, yet garlic extract at 500 mg/day has consistently been shown to reduce blood pressure (Appel 2017; Adegbola et al. 2017). This difference is due to differences in bioavailability; for example, resveratrol and other compounds have nonlinear dose–response curves (Haseeb et al. 2017). Careful consideration should be given to safety issues. Azadbakht (2007) stated that soy isoflavones, a phytoestrogen, may increase the international normalized ratio (INR) levels in warfarin patients by 1.5 points.

Di Minno et al. (2011) reported that grapefruit chemicals inhibit cytochrome P450 3A4, potentially resulting in a three to five‐fold increase in the blood concentration of calcium channel blockers. A comprehensive assessment of drugs is required before recommending phytochemical supplements because of these interactions (Stanner et al. 2019). Other phytochemicals have also been reported to significantly alter drug metabolism through their action on cytochrome P450 enzymes, P‐glycoprotein transporters, and hepatic conjugation processes. For example, flavonoids contained in grapefruit induce inhibition of CYP3A4, leading to increased plasma levels of statins such as simvastatin and atorvastatin, which can increase the risk of myopathy and rhabdomyolysis (Kondža et al. 2024). Similarly, garlic and *Ginkgo biloba* have been associated with augmented anticoagulant effects when taken with warfarin, increasing the bleeding risk by additive platelet inhibition and INR elevation (Soyata et al. 2020). Curcumin and green tea catechins also influence drug transport and metabolism and therefore should be used with caution in polypharmacy patients (Kyriacou et al. 2025). They emphasize the need for tailored assessments, especially in patients with cardiovascular comorbidities on chronic statin or anticoagulant therapy. Issues with standardization remain because of inherent variability. Green tea polyphenol content can vary by a factor of ten depending on the processing and cultivation conditions (Yang et al. 2007). Products available on the market usually have active compounds 20%–30% less than those claimed (Dwyer et al. 2018). Nussbaumer et al. (2011) observed that to ensure product uniformity, sophisticated analytical technologies like HPLC‐MS are needed. Therefore, phytochemicals may interact with widely used medications, such as anticoagulants (e.g., warfarin), antihypertensives (e.g., calcium channel blockers), and statins, requiring vigilant monitoring and individualized evaluation prior to clinical application (Jyothi et al. 2025).

### 7.3 Phytochemical‐Based Nutraceuticals in CVD Management

Innovative nutraceutical blends break the longstanding limits. As reported by Pala et al. (2020), nano‐encapsulation increases the bioavailability of resveratrol from < 1% to > 40%, allowing therapeutic doses of100 to 2000 mg per day to be effectively used. Combination products, such as CardioAid (berberine, red yeast rice, and coenzyme Q10), have been shown by clinical trials to lower LDL by as much as low‐dose statins (Altamimi et al. 2020). The use of medical foods is another promising approach. In addition to statins, the FDA‐approved plant sterol CoroWise can lower LDL cholesterol by an additional 10% to 155% (Guasch‐Ferré et al. 2018). Similarly, based on Martínez‐González et al. (2019), endothelial function was found to be 28% improved when taking flavonoid‐enriched Mediterranean plant powder (FMPP) over standard dietary advice. Future research could involve the creation of pharmacogenomics‐based personalized nutraceuticals. Flavonoid dosing in accordance with the CYP2C9 genotype can be useful for patients taking warfarin, as indicated by Alradwan et al. (2024). Likewise, gut microbiome characterization can be utilized to more precisely target microbial metabolic patterns via prebiotic‐phytochemical combinations (Gamalero and Glick 2022). Although there is much potential for the amalgamation of phytochemicals with conventional cardiovascular treatment, this method must be standardized and tested for safety (Dwyer et al. 2018). Alradwan et al. (2024) stated that the current gaps between conventional medicine and evidence‐based phytotherapy might be bridged by personalized methods and novel nutraceutical technology. Haines et al. (2024) emphasized that clinical trials comparing combination therapies, such as phytochemical adjuvants, were a research priority to determine efficacy without risking potential side effects. Special attention should be given to pharmacokinetic interactions and long‐term safety findings. Adjuvant phytochemical‐derived compounds may benefit whole strategies for decreasing cardiovascular risk.

## 8 Conclusion and Future Perspectives

One likely and multifaceted method for the prevention and treatment of cardiovascular diseases is the intake of dietary phytochemicals. These are key elements of cardioprotective treatments because they can address significant pathological mechanisms, such as inflammation, oxidative stress, dyslipidemia, endothelial dysfunction, and hypertension. These chemicals, which are abundant in fruits, vegetables, nuts, and teas, offer nutritional and medicinal value, especially as part of a healthy diet such as the Mediterranean diet. The therapeutic potential of phytochemicals in diverse populations, including encouraging evidence from preclinical and observational studies, must be confirmed by long‐term clinical trials. However, most of these studies rely on in vitro testing or animal models, which may not accurately represent human physiology. Furthermore, doses used in study designs are generally higher than normal dietary consumption, and intervention durations are usually too short to show evidence of long‐term cardiovascular impact. To fully realize their potential, it is essential to standardize dosages, enhance our knowledge of bioavailability, and combine them with conventional pharmacological therapies. Despite progress, key gaps remain in current research. Firstly, missing are universal formulations and dosing guidelines for phytochemical‐based interventions to limit their clinical efficacy. Second, little is understood about the role of individual‐level determinants, such as genetic heterogeneity, gut microbiota composition, and metabolic rate, in affecting the bioavailability and efficacy of phytochemicals. Third, few large‐scale, long‐duration randomized controlled trials of representative populations have been conducted, which limits the generalizability of the current evidence. In summary, phytochemicals possess great potential as an addition to existing treatment programs, a key to reducing the global burden of cardiovascular disease, and a defender of improved cardiac health in the future.

The specific molecular targets and signaling pathways that phytochemicals employ to affect heart function will be the focus of future research. One step towards creating personalized dietary regimens is to learn how these compounds interact with individual genetic profiles, metabolic rates, and gut microbiota. In addition, phytochemicals can become more clinically relevant owing to advances in food science and delivery methods that enhance their bioavailability and therapeutic predictability. To translate phytochemical research into actionable suggestions and therapeutic practice, multidisciplinary collaboration among physicians, pharmacologists, and dietitians is needed at the earliest opportunity. To assess the long‐term safety and efficacy of phytochemicals, randomized controlled trials involving standardized preparations, confirmed biomarkers, and heterogeneous cohorts are necessary. One long‐term, ecologically safe approach to address the global burden of cardiovascular disease is to add phytochemicals to public health policy and prevention medicine regimens. However, if repeated verification by future evidence confirms the cardioprotective activities of phytochemicals, public health guidelines may be revised to offer precise daily intake recommendations for bioactive compounds, such as flavonoids and polyphenols. Dietary policies at the national level would also promote the inclusion of phytochemical‐rich foods such as berries, leafy greens, nuts, and tea as part of cardiovascular prevention programs. Tailored nutritional advice can also be derived, advising health practitioners on how to tailor phytochemical‐dietary advice based on the risk level and hereditary tendencies of an individual.

## Author Contributions

**Muhammad Tayyab Arshad:** writing – original draft (equal). **M. K. M. Ali:** data curation (equal), methodology (equal). **Sammra Maqsood:** writing – review and editing (equal). **Ali Ikram:** supervision (equal). **Md. Sakhawot Hossain:** data curation (equal), formal analysis (equal). **A. I. Aljameel:** data curation (equal), visualization (equal). **Ammar AL‐Farga:** data curation (equal), investigation (equal). **Kodjo Théodore Gnedeka:** validation (equal).

## Disclosure

The authors have nothing to report.

## Ethics Statement

This study did not involve humans or animals.

## Consent

This study did not involve humans.

## Conflicts of Interest

The authors declare no conflicts of interest.

## Data Availability

Data supporting the findings of this study are available from the corresponding author upon reasonable request.
